# Antilisterial Effect of a Natural Formulation Based on Citrus Extract in Ready-To-Eat Foods

**DOI:** 10.3390/foods10071475

**Published:** 2021-06-25

**Authors:** Juan José Ariza, David García-López, Esperanza Sánchez-Nieto, Enrique Guillamón, Alberto Baños, Manuel Martínez-Bueno

**Affiliations:** 1 DMC Research Center, Camino de Jayena, 82, 18620 Alhendín, Spain; jariza@dmcrc.com (J.J.A.); dgarcia@dmcrc.com (D.G.-L.); espe.sanchez2@gmail.com (E.S.-N.); eguillamon@domca.com (E.G.); 2 Departamento de Microbiología, Universidad de Granada, Avda. Fuentenueva, s/n, 18071 Granada, Spain; mmartine@ugr.es

**Keywords:** *Listeria*, natural preservatives, flavonoids, food safety

## Abstract

Controlling *Listeria* in food is a major challenge, especially because it can persist for years in food processing plants. The best option to control this pathogen is the implementation of effective cleaning and disinfection procedures that guarantee the safety and quality of the final products. In addition, consumer trends are changing, being more aware of the importance of food safety and demanding natural foods, minimally processed and free of chemical additives. For this reason, the current consumption model is focusing on the development of preservatives of natural origin, from plants or microorganisms. In sum, this study aimed to evaluate the antimicrobial effectiveness of a citrus extract formulation rich in flavonoids against several *L. monocytogenes* and *L. innocua* strains, using in vitro test (agar diffusion test, minimum bactericidal concentration (MBC), and time-kill curves) and challenge test in food trials (*carne mechada*, salami, fresh salmon, lettuce, brine, and mozzarella cheese). The results presented in this work show that citrus extract, at doses of 5 and 10%, had a relevant antimicrobial activity in vitro against the target strains tested. Besides this, citrus extract applied on the surface of food had a significant antilisterial activity, mainly in *carne mechada* and mozzarella cheese, with reductions of up to eight logarithmic units with respect to the control. These results suggest that citrus extract can be considered a promising tool to improve the hygienic quality of ready-to-eat foods.

## 1. Introduction

Foodborne diseases are a reality affecting thousands of people in industrialized countries every year. According to the report published by EFSA in 2019 “The European Union One Health 2018 Zoonoses Report” [1], the trend of food and waterborne outbreaks have remained constant since 2014. These were caused by *Campylobacter jejuni*, *Listeria monocytogenes*, *Salmonella enterica*, or verotoxigenic *Escherichia coli*, among others. In recent years, a growing trend has been observed in the number of outbreaks caused by *L. monocytogenes*. Indeed, it is considered one of the most serious zoonosis, forward by *C. jejuni* and *S. enterica*, causing the highest hospitalization and fatality rates (25–30%, similar to other pathogens such as *Salmonella*) [2,3], particularly in young, old, pregnant, and immunosuppressed (YOPI) consumers. The food vehicles with the highest impact analyzed have been dairy products, followed by meat, fishery products, and RTE (ready to eat) foods [4], due to their origin and the way in which they are processed. In fact, cold-smoked fish and RTE meat products were the most significant cause of *Listeria*-associated outbreaks in 2019 [5]. In addition, there was an increase in outbreaks observed triggered by the intake of fresh-cut fruits and vegetables [6].

This increase in *Listeria* infections in recent years can be explained by aging of the population and the changes in consumer habits. This change of dietary models has led European consumers to prefer natural foods, less industrially processed and free of synthetic additives. For this reason, the current consumption trend is focusing on developing natural preservatives with antimicrobial activity from plants and microorganisms in order to replace traditional chemical treatments (sorbates, benzoates, nitrates, etc.) [7].

Among the natural preservatives currently studied, plant extracts such as essential oils (EOs) have been proved to host a wide spectrum of compounds with antimicrobial activity (against pathogens as *L. monocytogenes*) and interesting antioxidant properties [8,9,10]. Some authors have shown that many EOs, such as oregano, thymol, cloves, or basil, have a GRAS status (Generally Recognized As Safe), allowing their use as natural preservatives in food [11]. In addition, thymol, eugenol, carvacrol, cinnamaldehyde, or limonene have been accepted in Europe with the tag of flavoring ingredients, as they do not present a health risk for consumers, [12].

Citrus extracts, rich in flavonoids, are natural products containing a variable number of phenolic groups in their structure, which gives them a great antioxidant capacity [9]. Moreover, flavonoids such as hesperidin or naringin have been shown to be compounds with a high capacity for peroxidative protection, with important antimicrobial action even able to eliminate *L. monocytogenes* biofilms [13]. These products can damage both the cell wall and membrane and acidify the cell cytoplasm, causing irreversible lethal damage [14].

To sum up, this study aims to evaluate the antimicrobial effectiveness of a citrus extract formulation rich in flavonoids against several *L. monocytogenes* and *L. innocua* strains, using both in vitro and food trials.

## 2. Materials and Methods

### 2.1. Bacterial Strains and Used Growth Media

The microorganisms used for this study were obtained from the CECT (Spanish Collection of Type Cultures) and wild isolates from our collection (DMC Research) (Table 1). Concerning wild strains, they were originally isolated from the food industry and stored with 20% glycerol at −70 °C. The culture media used were BHA (brain heart agar) buffered for antibiosis tests, and chromogenic culture medium Compass *Listeria* and the Fraser broth pre-enrichment medium, supplied by Biokar Diagnostics (Allone, France) previously prepared and autoclaved, for the selective growth of *Listeria* spp. for in vitro and challenge tests.

### 2.2. Citrus Extract

A natural formulation based on the combination of a citrus extract (20%) obtained from bitter orange (*Citrus aurantium*) along with organic acids used as stabilizers and obtained by fermentation (5% lactic acid and 4% citric acid) was provided by DOMCA S.A.U. (CYCROM PRO DMC^®^, Granada, Spain). This citrus extract contained flavoring compounds, including flavonoids (especially hesperidin, naringin, naringenin, and apigenin), alkaloids (p-synephrine), and monoterpene hydrocarbons (mainly d-limonene).

### 2.3. In Vitro Tests for the Evaluation of the Antimicrobial Activity

Different in vitro tests (agar diffusion test, inhibition test in liquid medium, minimum bactericidal concentration, and time-kill curves) were performed to determine the antimicrobial efficacy of citrus extract.

For the agar diffusion test [15], bacterial cultures of different *Listeria* strains were incubated at 37 °C for 24 h. The inhibition zone of bacterial growth is proportional to the degree of inhibition, produced by a sterilized cellulose disc (6 mm Whatman^®^ antibiotic test discs, Buckinghamshire, UK) impregnated with 20 µL of pure citrus extract. 

The method proposed by Tagg and McGiven [15] was carried out to determine the antimicrobial activity of the product in liquid medium. Stainless steel towers of 8 mm diameter × 10 mm high (Stainless Steel Cilinders for Antibiotic, Scharlab) were placed on BHA-buffered plates, and an overlay of BHA at 45 °C was added, previously inoculated with a concentration of 8 Log_10_ CFU/mL of the target strain. The steel towers were removed once the overlay had solidified, and 100 µL of citrus extract was added and incubated at 37 °C for 18–24 h. The activity was expressed by the diameter of the inhibition zone (mm). 

The MBC (minimum bactericidal concentration) was used to determine the lowest concentration of an antimicrobial agent that reduces the viability of the initial bacterial inoculum by 99.9%. The method used for this study was broth microdilution according to the National Committee for Clinical Laboratory Standards [16]. Decreasing concentrations (25,000, 12,500, 6250, 2500, 3125, 1562.5, 1000, 781,025, 50, 390.625, 250, 125, and 62.5 mg/L) of citrus extract were used and inoculated with different bacterial strains to obtain a final concentration of 5 Log_10_ CFU/mL. For the positive control, a well with a concentration of nisin (125 IU/mL) was used [17]. As negative control, another well without bacteria or any antimicrobial agent was used. Finally, every well with no cell growth (measured by absorbance at 620 nm) was tested by being cultured in selective agar plates and incubated at 37 °C for 24 h, in order to determine the MBC.

Time-kill curves were performed following the procedure described by Guerrillot et al. [18]. Different concentrations of citrus extract (0, 0.5, 1, 5, and 10%) were evaluated in buffered peptone water, starting from an initial bacterial inoculum adjusted to 6–8 Log_10_ CFU/mL (depending on the strain). Samples were collected at different time intervals (0, 30, 60, 120 min, and 6 and 24 h), plated on a selective agar medium, and incubated at 37 °C for 24–48 h. Time-kill curves showed the results of Log_10_ CFU/mL versus time. The detection limit of counting methods used was 0.3 Log_10_ CFU/mL. 

All in vitro assays were performed in duplicate.

### 2.4. Challenge Tests

Antilisterial efficacy of citrus extract was evaluated in different food models, whose process of elaboration is described below:

- The *carne mechada* samples were prepared from fresh pig head supplied by a local butcher shop (Alhendín, Spain). The piece of meat was cleaned, weighted, and cut. For the preparation of the brine, we solved 105 g in water of commercial preparation composed of salt, maltodextrin, corn starch, and sodium ascorbate (ref. 110249, from the DOMCA trademark, Granada, Spain). Then, the brine was injected inside the meat piece using a roving machine with an individual injector (Suministros Lizondo, Barcelona, Spain). The meat was then transferred to the mixing drum (Mixer RM-20, MAINCA SL, Barcelona, Spain). A potato starch solution (5%) was added and subsequently homogenized for 15 min. Then, the piece was manually stuffered (EC-12, MAINCA SL) in polyamide casing (FIBRACO, Barcelona, Spain) and finally cooked at 70–75 °C for 2 h. After 24 h, the meat was cut into 6–8 mm thickness slices, which were then divided into expanded polystyrene trays (135 × 80 mm, Bandesur SA, Jaén, Spain). Afterwards, these were vacuumed (Tecnotrip EVT-10–2-CV-SC, Barcelona, Spain) for further storage in refrigeration.
- Salami samples were prepared from pork supplied by a local butcher (Alhendín, Spain). Previously, 600 g of lean were frozen at −6 °C and chopped in a meat grinder (CUTTER (MAINCA SL) at 1500 rpm. A total of 130 g of ice and 70 g/kg of a commercial formulation based on salt, lactose, dextrin, pepper flavor, meat flavor, sodium ascorbate, and red dye were then added (ref. 70170162, DOMCA, Granada, Spain) and minced at 300 rpm. A total of 200 g/kg of frozen bacon was added and minced at 1500 rpm until a uniform dough was obtained. After that, this mixture was stuffed using a manual casing stuffer (FIBRAN) and refrigerated at 4–6 °C for 8–10 days, with a humidity of 90–95%. The sausage was then stored in the drying room for 10 days at 12–15 °C and humidity of 75%. Before use, salami was cut into 6–8 mm thickness slices.
- The piece of fresh salmon (*Salmo salar*) was supplied by a local fishmonger (Alhendín, Spain). It was cleaned by removing excess fat and cut into symmetrical heavy pieces of 5 × 5 cm (25 cm^2^).
- Lettuce of the iceberg variety was supplied by a local fruit store (Alhendín, Spain). The leaves were washed with sterile water and cut into symmetrical pieces of 5 × 5 cm (25 cm^2^).
- Home-made mozzarella cheese pearls made of buffalo milk were supplied by a local store (Alhendín, Spain). The original brine was discarded and a brine with sterile water at 10% NaCl was added. After applying treatments to the brines and their subsequent homogenization, we added 20 mozzarella pearls per batch and stored them at 4 °C for 25 days.

Challenge test assays were performed according to Baños et al. [19]. *Carne mechada*, salami, lettuce, and salmon were inoculated with an adjusted concentration of 5 Log_10_ CFU/mL to obtain a final 2–3 Log_10_ CFU/cm^2^ concentration from a pool of *L. monocytogenes*, using a sterile handle Digralsky. Afterwards, different treatments (400 µL/25 cm^2^ of 0.5, 1, 5, and 10% citrus extract) were superficially sprayed using an automated spray system (AUTOJET 1550, Spraying System Co., Glendale Heights, IL, USA) without further rinsing. A distilled water spray treatment was carried out as control. The samples were individually packed in polystyrene trays and immediately sealed in Ziplock bags and vacuumed for their storage at 4 °C. In the case of mozzarella, *L. monocytogenes* was inoculated directly in the brine at a concentration of 2–3 Log_10_ CFU/mL. Two independent experiments were carried out for each food.

### 2.5. Monitoring

For the analysis of each food, samples were collected at different time intervals, according to the shelf life of each food matrix. For this, 1:10 dilutions of each food were made with buffered peptone water (Biokar Diagnostics), which were processed using a MASTICATOR mixer (IUL, Barcelona, Spain). The culture was performed on selective culture media plates and then incubated at 37 °C for 24–48 h. Results were expressed as Log_10_ CFU/cm^2^ versus time and Log_10_ CFU/g in the case of mozzarella. When it was not possible to quantify the bacteria below the detection limit (<1 Log_10_ CFU/cm^2^, <0.3 Log_10_ CFU/mL in brine y < 1 Log_10_ CFU/g in mozzarella), an investigation was carried out by pre-enrichment in Fraser broth (Biokar Diagnostics), expressing the results as presence or absence.

### 2.6. Statistical Analysis

The statistics were extracted from the results of two independent experiments. In each one, 3 samples (food tested) for each treatment (*n* = 6) and sampling time were used. The average data ± standard deviations were determined with Excel software (Microsoft Corp., Redmond, WA, USA). Statistical analyses were performed using the SPSS-PC 15.0 software (SPSS, Chicago, IL, USA). Data on microbiological counts were subjected to ANOVA. Error probability values less than 0.05 were considered not significant.

## 3. Results

### 3.1. In Vitro Tests to Evaluate Antimicrobial Activity

The antimicrobial efficacy of a formulation based on citrus extract was tested against different strains of interest of *Listeria* spp. (Table 1) involved in most of the routes of cross-contamination of surfaces in contact with food [20]. Table 2 shows the results of the diffusion test in agar and antibiosis in a liquid medium expressed as the diameter of the inhibition zone.

Most of the *Listeria* strains were sensitive to the citrus extract product. *L. innocua* DMC 5-1 and *L. innocua* DMC 6-2 were the most resistant strains, with smallest inhibition zones among the target strains studied. Table 2 also details the results obtained in the MBC tests, proving that the treatment reduces microbial counts below the detection limit (0.3 Log_10_ CFU/mL). Again, *L. innocua* DMC 5-1 was the most resistant strain, requiring high concentrations of citrus extract to reduce the viability of the initial bacterial inoculums.

**Figure 1:**
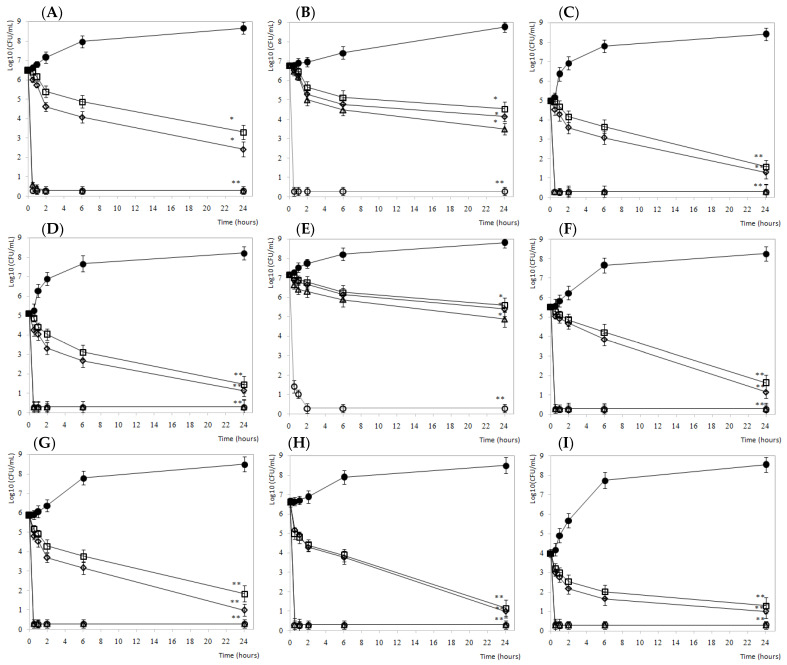
Microbial viability evolution of (**A**) *L. monocytogenes* CECT 4032; (**B**) *L. monocytogenes* CECT 5366, (**C**) *L. monocytogenes* DMC 1-23, (**D**) *L. monocytogenes* DMC 3-17, (**E**) *L. innocua* CECT 4030, (**F**) *L. innocua* DMC 4, (**G**) *L. innocua* DMC 5-1, (**H**) *L. innocua* DMC 6-2, and (**I**) *L. innocua* DMC 7-3 in the presence of citrus extract. Concentrations tested for each strain: ● 0 ppm (negative control), □ 0.5%, ♦ 1%, Δ 5%, and ○ 10% of citrus extract. Values are means with SD in bars. * *p* < 0.05; ** *p* < 0.001 respect to control.

Finally, to complete the in vitro studies, we performed a nutrient broth reduction test (time-kill curves). The results obtained are represented in the following Figure 1.

Time-kill curves provide additional information on the relationship between the concentration of the antimicrobial agent and its bactericidal activity, represented as Log_10_ CFU/mL as a function of time. All bacterial strains tested were susceptible to the formulation based on citrus extract. Figure 1 shows a decrease in the initial cell concentration as a function of time at different product concentrations (0.5, 1, 5, and 10%). Results showed a bactericidal effect at 24 h when testing 5 and 10% of citrus extract (*p* < 0.001). Likewise, a significant antilisteria effect (*p* < 0.05) of the treatment at the doses of 0.5% and 1% against all the strains studied was observed. As an exception, for the most resistant strains of those studied, *L. monocytogenes* CECT 5366 and *L. innocua* CECT 4030, only a bactericidal effect (*p* < 0.001) was achieved at a concentration of 10% of citrus extract. These strains require a longer exposure over time and a higher concentration of disinfectant than for the complete inactivation than other strains. Besides this, it was observed that at 5 and 10% of citrus extract total disinfection (<0.3 Log_10_ CFU/mL) was achieved between 30 min and 2 h of contact.

### 3.2. Efficacy Evaluation Trials in Food

Currently, foodborne diseases continue to be one of the most important public health problems, both in developed and developing countries. According to the WHO, around 600 million people worldwide fall ill each year from foodborne diseases. These numbers confirm the need to develop new conservation methods, such as natural plant extracts, that do not promote the appearance of microbial resistance arising from the continued use of traditional chemical preservatives [21].

After verifying the antimicrobial activity of citrus extract by in vitro tests, we performed effectiveness tests in food against a pool of *L. monocytogenes* strains.

#### 3.2.1. Carne Mechada

**Figure 2:**
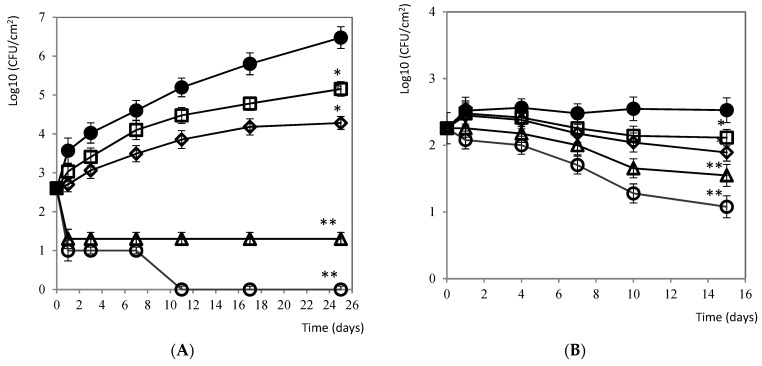
(**A**) Evolution of *L. monocytogenes* on the surface of *carne mechada* fillets during 25 days of conservation at 4 °C against the different concentrations tested: ● 0 ppm (negative control), □ 0.5%, ♦ 1%, Δ 5% y, and ○ 10% of citrus extract. Values are means with SD in bars. * *p* <0.05; ** *p* <0.001 with respect to the control. (**B**) Evolution of *L. monocytogenes* on the surface of salami during 15 days of conservation at 4 °C against the different concentrations tested: ● 0 ppm (negative control), □ 0.5%, ♦ 1%, Δ 5% y, and ○ 10% of citrus extract. Values are means with SD in bars. * *p* < 0.05; ** *p* < 0.01 with respect to the control.

Figure 2A shows the difference between the control batch and the treatments from the beginning of the trial. From the start of its treatment, citrus extract at doses of 5 and 10% were effective against the target *Listeria* strains studied, with significant reductions (*p* < 0.001) 25 days after the trial. In addition, 7 days after applying the 10% of citrus extract, the pathogen was completely eliminated. Doses of 0.5 and 1% of the product achieved reductions up to 2 units of difference (*p* < 0.05) with respect to the control after 25 days of preservation.

#### 3.2.2. Salami

The treatment of salami surface with citrus extract (5 and 10%) were the most effective against *Listeria*, with significant reductions of the pathogen (*p* < 0.01) after 15 days of refrigerated storage. Doses of 0.5 and 1% of the product reduced the population of *Listeria* up to a half logarithmic unit of difference (*p* < 0.05) with respect to the control until the end of the study (Figure 2B).

#### 3.2.3. Fresh Salmon

**Figure 3:**
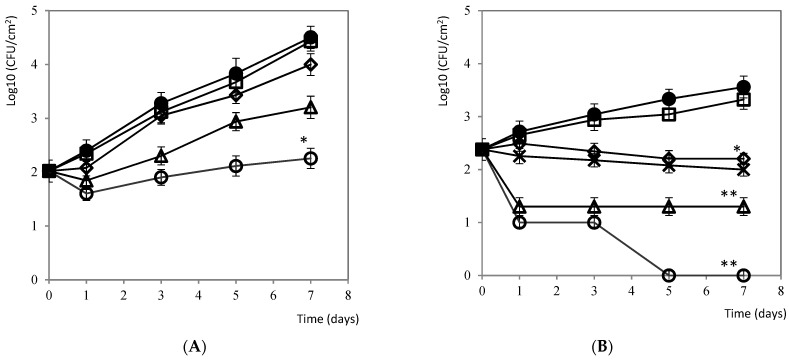
(**A**) Evolution of *L. monocytogenes* on the surface of salmon during 7 days of conservation at 4 °C: ● 0 ppm (negative control), □ 0.5%, ♦ 1%, Δ 5% y, and ○ 10% of citrus extract. Values are means with SD in bars. * *p* < 0.05 with respect to the control. (**B**) Evolution of *L. monocytogenes* on the surface of lettuce during 7 days of conservation at 4 °C: ● 0 ppm (negative control), □ 0.5%, ♦ 1%, Δ 5% y, and ○ 10% of citrus extract; X 150 mg/L of sodium hypochlorite. Values are means with SD in bars. * *p* < 0.01; ** *p* < 0.001 with respect to the control.

*L. monocytogenes* could be implanted without problems on the surface of the salmon samples during the 7 days of preservation at 4 °C (Figure 3A). Spraying with citrus extract at 10% had a significant effect (*p* < 0.05) compared with the untreated control after 7 days. This effect was particularly remarkable at the end of the assay, with a difference of 2 Log_10_ CFU/cm^2^ (*p* < 0.01) with respect to the control treatment.

#### 3.2.4. Lettuce

The use of disinfectants, such as sodium hypochlorite, to wash minimally processed vegetables is authorized for the purpose of delaying or eliminating the growth of pathogenic microorganisms. Although it is economically viable, the formation of trihalomethanes (carcinogenic compounds) requires the search for new natural preservatives as an alternative to these chemical disinfectants [22]. The microbiological shelf life of lettuce, previously washed with sodium hypochlorite (150 mg/L), is approximately 7 to 10 days, stored at 4 °C [23]. In this study, the use of citrus extracts at doses of 5 and 10% on the surface of lettuce leaves could be considered as a natural alternative to the traditional use of sodium hypochlorite, with significant microbial reductions compared to the control group (*p* < 0.001), and with respect to the chemical treatment (*p* < 0.05) (Figure 3B). Moreover, at the dose of 1% of citrus extract, similar reductions to those obtained with 150 ppm of sodium hypochlorite were observed (*p* < 0.01).

#### 3.2.5. Mozzarella Cheese and Brine

**Figure 4:**
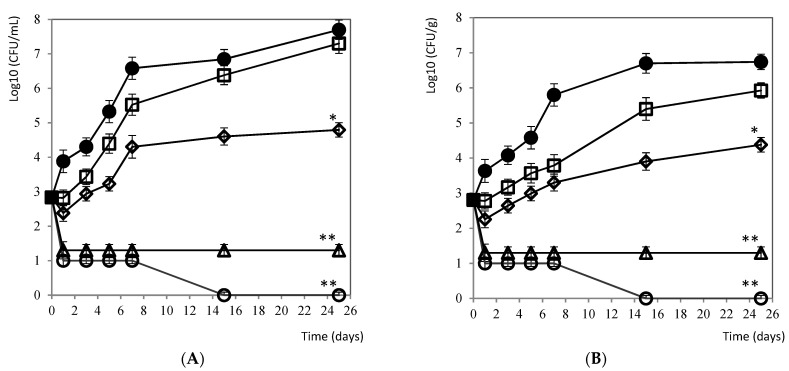
(**A**) Evolution of *L. monocytogenes* in (**A**) mozzarella cheese and (**B**) brine during 25 days of conservation at 4 °C: ● 0 ppm (negative control), □ 0.5%, ♦ 1%, Δ 5%, and ○ 10% of citrus extract. Values are means with SD in bars. * *p* < 0.05; ** *p* < 0.001 with respect to the control.

The antimicrobial effectiveness of citrus extract at different doses in brine and in mozzarella cheese are shown in Figure 4A,B. Significant bacterial reductions (*p* < 0.001) with respect to the control were observed from the beginning of the trial at doses of 5% and 10% of citrus extract. This effect was maintained after 25 days of preservation. Seven days after applying the 10% treatment, the pathogen was completely eliminated, both in mozzarella and brine. Although to a lesser extent, the application of 1% of the treatment produced significant reductions (*p* < 0.05) of *L. monocytogenes* after 15 days of storage, both in mozzarella and in brine.

## 4. Discussion

The interest in the assessment of the antimicrobial activity of citrus extract as a natural preservative is related to the increasing trend that has been observed in recent years in the number of foodborne diseases caused by *L. monocytogenes*. This antimicrobial efficacy of a citrus extract has been tested against different strains of interest in the food industry such as *Listeria* spp. when used as natural food preservatives [24]. The results showed some differences in the MBC values obtained, even for different species within the same genus. This may be due to the fact that the sensitivity or resistance to a certain antimicrobial agent is a property that is established at the strain level, since the defense mechanisms against a specific substance can vary between different strains of the same species [25].

The formulation used in these trials bases its activity on the presence of citrus extracts rich in flavoring compounds such as flavonoids. As reported by Barreca et al. [26], flavonoids such as hesperetin and hesperidin exert a greater inhibitory activity in vitro against Gram-negative bacteria, whilst another research has shown that the antimicrobial activity of naringin and its derivatives is greater against Gram-positive such as *Listeria* spp. Although the mechanism of action of compounds rich in flavonoids is not clear, different mechanisms have been proposed such as the rupture of the bacterial membrane, modifications in the permeability of the cell wall, or the interference in the synthesis of microbial DNA [27].

After verifying the antimicrobial activity of citrus extract by in vitro tests, efficacy tests were performed in different trial food, such as *carne mechada*, salami, fresh salmon, lettuce, brine, and mozzarella cheese.

*Carne mechada* is a typical Andalusian dish consisting of pork head meat that is consumed cut into slices. It is a pre-cooked and ready-to-eat product that, in most cases, will not undergo any heat treatment before being consumed. In 2019, Spain suffered the most important listeriosis outbreak in its history due to the consumption of different batches of contaminated *carne mechada*. The European regulation on microbiological criteria [28] sets a limit of “Absence in 25 g of *L. monocytogenes*” in ready-to-eat foods, ensuring that this limit is not exceeded throughout the shelf life of the food. In view of the results, citrus extract applied at 5 and 10% on the surface of *carne mechada* could contribute the control of this pathogen under this parametric limit during 25 days of product conservation. Furthermore, there is already evidence in previous studies that citrus extracts, rich in flavonoids, are postulated as a natural alternative to the use of chemical preservatives in the control of this pathogen in meat matrices [29]. Mhalla et al. [30] reached this conclusion when evaluating fractions of a plant matrix rich in flavonoids, using them as natural preservatives in the control of *L. monocytogenes* in minced beef for 30 days at 4 °C. Other studies also proved the important antimicrobial activity of these botanical plants (rich in flavonoids) against *L. monocytogenes*, delving into the mechanism of action, since they act as inhibitors of bacterial growth or cell viability. This efficacy was demonstrated in ready-to-eat raw and processed minced chicken feed models [27,31]. In cured meat products such as salami the reduction of more than 25% of *Listeria* with respect to the control can be compared with the results obtained by Dussault et al. [32], who obtained reductions of *L. monocytogenes* of between 10% and 19% through the use of natural preservatives such as essential oils.

In addition, the efficacy of citrus extract has been evaluated in the food vehicles most susceptible to being contaminated by *L. monocytogenes*, such as vegetables and fishery and dairy products RTE.

In the trial carried out in fresh salmon, the growth of *L. monocytogenes* observed in all treated samples during the storage can be attributed to the adsorption to the food matrix and to the recovery of sublethally damaged *Listeria* [33]. The addition of organic acids in smoked salmon, such as acetic or lactic acid, has proven to be an effective strategy against the control of *L. monocytogenes* to rise the values approved by the EU regulation on RTE (not exceeding the critical limit of 2 Log_10_ CFU/g) [34]. There are few references on the potential use of citroflavonoids in the control of fish pathogens; however, the antimicrobial efficacy of different plant extracts for the control of *L. monocytogenes* in smoked salmon has been evaluated as *Cinnamom umjavanicum* plant [35]. In addition, the food industry has been interested in the development of innovative biomaterials with antimicrobial properties, such as the use of polyphenolic coatings obtained from plant extracts. These coatings have been applied to fresh salmon pieces to control *L. monocytogenes* during refrigeration [36].

The test carried out on lettuce showed one of the most satisfactory results—even applying just 10% of the treatment, a total reduction of the pathogen was achieved on the fifth day of sampling, with “absence” results after pre-enrichment in Fraser broth and subsequent plate culture. These results can be contrasted with those obtained by Yu et al. [37], who demonstrated the efficacy of citrus extracts obtained from grapefruit against *L. monocytogenes* in lettuce. Vázquez-Armenta et al. [38] also obtained similar results in the tests carried out on lettuce leaves using a flavonoid-rich grape extract as a natural preservative, with microbial reductions of 1.8 logarithmic units in the case of *E. coli* and 0.86 logarithmic units of *L. monocytogenes*. Results demonstrated that citrus extract is a good option as a natural sanitizer for vegetables and an alternative to the use of sodium hypochlorite as a traditional chemical disinfectant.

Furthermore, brines of dairy industry are susceptible to microbiological contamination, with the use of chemical sanitizing treatments based on hydrogen peroxide [39] or chlorine dioxideto control of *L. monocytogenes* being common, with bacterial reductions of up to four logarithmic units [40]. In this case, the addition of citrus extract can be even more effective, with reductions of up to seven logarithmic units from the start of the trial. For this reason, this natural treatment could be postulated as a natural alternative to the use of chemical additives in brines.

Cheese contamination with foodborne bacterial pathogens is a serious problem that could be caused by various sources during cheese production or storage. Traditionally, many plants and their derivatives have been used as possible natural antimicrobial alternatives for the preservation of these foods. Plant extracts, commonly used as spices and flavoring agents of cinnamon, cloves, oregano or lemon, have shown an important antimicrobial efficacy against the control of pathogens such as *L. monocytogenes* or *Staphylococcus aureus*, in addition to sensory improvement of cheeses [41,42]. This antilisterial effect has also been proven in cheeses of Italian origin such as ricotta [43], demonstrating the potential of natural extracts as natural preservatives of these dairy products. Finally, the antimicrobial efficacy of citroflavonoids has been proven when incorporated into cheese maturing brines and also when applied directly to the surface of cheeses in the form of chitosan polymer biofilms, achieving an important bacteriostatic effect against pathogens of interest in the dairy industry such as *E. coli* or *Aspergillus niger* [44].

## 5. Conclusions

The antilisterial effectiveness of a food-grade product based on citrus extract as a natural preservative has been tested both in vitro tests and challenge tests. The results presented in this study show the ability of citrus extract to control several strains of *L. monocytogenes* and *L. innocua*. Although further efficacy trials are needed, we consider that this natural solution is a promising tool to improve the hygienic quality and food safety of ready-to-eat food, mainly in *carne mechada*, mozzarella cheese, and lettuce.

## Figures and Tables

**Table 1:** References and uses of the strains used.

**Strain Used for Vitro Test and Challenge Test**	**Isolated**
*Listeria monocytogenes* CECT 4032	Clinical isolate of meningitis
*Listeria monocytogenes* CECT 5366	Clinical isolate
*Listeria monocytogenes* DMC 1-23	Minced meat isolated
*Listeria monocytogenes* DMC 3-17	Fresh cheese isolated
**Strain Used for In Vitro Test**	**Isolated**
*Listeria innocua* CECT 4030	Fresh cheese isolated
*Listeria innocua* DMC 4	Minced meat isolated
*Listeria innocua* DMC 5-1	Fresh cheese isolated
*Listeria innocua* DMC 6-2	Food industry isolated
*Listeria innocua* DMC 7-3	Food industry isolated

**Table 2:** Results of the antibiosis tests on solid medium, minimum bactericidal concentration (MBC), and antimicrobial activity on liquid medium (Tagg and McGiven) of citrus extract against reference strains. The results with the positive control (nisin) are not shown.

Strain	MBC (mg/L)	Inhibition Zone (mm)	Activity on Liquid Medium (mm)
*Listeria monocytogenes* CECT 4032	5000	13 ± 1	15 ± 1
*Listeria monocytogenes* CECT 5366	5000	13 ± 1	13 ± 1
*Listeria monocytogenes* DMC 1-23	5000	12 ± 0	14 ± 1
*Listeria monocytogenes* DMC 3-17	5000	12 ± 0	14 ± 1
*Listeria innocua* CECT 4030	5000	13 ± 1	17 ± 1
*Listeria innocua* DMC 4	7812.5	12 ± 1	15 ± 0
*Listeria innocua* DMC 5-1	15,625	12 ± 1	13 ± 1
*Listeria innocua* DMC 6-2	7812.5	11 ± 2	13 ± 1
*Listeria innocua* DMC 7-3	5000	14 ± 1	15 ± 1
